# Modeling Inherited Disorders of Post-Lanosterol Cholesterol Biosynthesis: From Animal Models to Patient-Derived Stem Cells

**DOI:** 10.3390/ijms27156853

**Published:** 2026-07-30

**Authors:** Elvira Akhmetzyanova, Evelina Nasybullina, Albert Rizvanov, Yana Mukhamedshina

**Affiliations:** 1 OpenLab Gene and Cell Technology, Institute of Fundamental Medicine and Biology, Kazan Federal University, 420008 Kazan, Russiaalbert.rizvanov@kpfu.ru (A.R.); yomuhamedshina@kpfu.ru (Y.M.); 2 Division of Medical and Biological Sciences, Tatarstan Academy of Sciences, 420013 Kazan, Russia; 3 Department of Histology, Cytology, and Embryology, Kazan State Medical University, 420012 Kazan, Russia

**Keywords:** cholesterol biosynthesis, Smith–Lemli–Opitz syndrome, desmosterolosis, lathosterolosis, CHILD syndrome, DHCR7, DHCR24, SC5D, NSDHL, animal models, patient-derived fibroblasts, iPSCs, CRISPR/Cas9

## Abstract

Altered post-lanosterol cholesterol biosynthesis causes a heterogeneous group of rare inherited metabolic disorders, including Smith–Lemli–Opitz syndrome, desmosterolosis, lathosterolosis, and congenital hemidysplasia with ichthyosiform nevus and limb defects syndrome. These conditions are characterized by impaired cholesterol synthesis together with the accumulation of disease-specific sterol intermediates. Current experimental and clinical evidence suggests that pathogenesis reflects both cholesterol insufficiency and sterol-mediated toxicity, including oxidative stress, perturbed developmental signaling, membrane dysfunction, and impaired neurodevelopment. Experimental models have played a central role in elucidating these mechanisms and in evaluating emerging therapeutic strategies. This review provides a comprehensive overview of currently available experimental models used to investigate inherited cholesterol biosynthesis disorders, including genetically engineered animal models, patient-derived fibroblasts, immortalized and CRISPR/Cas9-edited cell lines, and induced pluripotent stem cell-based systems. Particular emphasis is placed on Smith–Lemli–Opitz syndrome, the most extensively studied disorder within this group, while recent advances in modeling desmosterolosis, lathosterolosis, and congenital hemidysplasia with ichthyosiform nevus and limb defects syndrome are also critically discussed. We compare the strengths and limitations of each experimental platform, highlighting their contributions to understanding sterol metabolism, developmental abnormalities, and cell-type-specific disease mechanisms. Finally, we discuss current challenges and future perspectives, including the development of patient-specific induced pluripotent stem cell models, genome editing approaches, and next-generation multicellular systems. Collectively, this review provides an updated framework for selecting appropriate experimental models to investigate cholesterol biosynthesis disorders and accelerate the development of mechanism-based therapeutic strategies.

## 1. Introduction

Cholesterol is an essential component of mammalian cells that performs diverse structural and regulatory functions beyond its well-established role as a membrane constituent. It serves as the precursor for steroid hormones, bile acids, and vitamin D, while also contributing to the formation of lipid rafts that regulate membrane protein organization, intracellular trafficking, and signal transduction. During embryonic development, cholesterol is particularly critical for the central nervous system, where it supports neural progenitor proliferation, neuronal differentiation, axonal guidance, synaptogenesis, and myelination. Moreover, cholesterol is indispensable for several developmental signaling pathways, including Sonic Hedgehog (Shh), Wnt/β-catenin, and Notch, explaining why disturbances in cholesterol homeostasis result in complex congenital malformations and neurodevelopmental abnormalities [1,2,3].

Inherited disorders of cholesterol biosynthesis comprise a heterogeneous group of rare genetic diseases caused by pathogenic variants in enzymes of the post-lanosterol cholesterol biosynthetic pathway. Among them, Smith–Lemli–Opitz syndrome (SLOS), desmosterolosis, lathosterolosis, and congenital hemidysplasia with ichthyosiform nevus and limb defects (CHILD) syndrome represent the best-characterized disorders associated with pathogenic variants in *DHCR7*, *DHCR24*, *SC5D*, and *NSDHL*, respectively. Despite affecting consecutive enzymatic reactions within the same metabolic pathway, these disorders exhibit remarkable clinical heterogeneity. The severity of clinical manifestations varies considerably even among patients carrying identical pathogenic variants, indicating that disease pathogenesis is influenced by multiple molecular mechanisms extending beyond simple cholesterol deficiency [4].

Historically, the clinical manifestations of cholesterol biosynthesis disorders were attributed primarily to insufficient cholesterol production. The available evidence now supports a more differentiated interpretation in which cholesterol deficiency and the accumulation of pathway-specific sterols contribute in proportions that depend on the affected enzyme, cell type, and developmental stage. In SLOS, 7-dehydrocholesterol (7-DHC) is exceptionally susceptible to oxidation, and 7-DHC-derived oxysterols have been linked to oxidative stress, altered membrane organization, and disrupted developmental signaling [3,5,6]. By contrast, desmosterol can partially substitute for cholesterol in some membrane contexts, whereas the cellular effects of lathosterol and methylsterols accumulating in lathosterolosis and CHILD syndrome remain less systematically characterized. These differences argue against treating all post-lanosterol disorders as a single mechanistic category and make disease-specific biochemical validation essential when selecting an experimental model.

Because these disorders are extremely rare and disease-relevant tissues, particularly the developing human brain, are largely inaccessible for detailed study, experimental models have become indispensable for investigating disease mechanisms and evaluating therapeutic strategies. Over the past three decades, disease modeling has evolved considerably, progressing from genetically engineered animal models and patient-derived fibroblasts to immortalized cell lines, CRISPR/Cas9-edited systems, and induced pluripotent stem cells (iPSC)-based platforms. Each model addresses distinct aspects of disease biology. Animal models provide systemic information regarding embryonic development, tissue pathology, and therapeutic efficacy but may incompletely reproduce human sterol metabolism because of species-specific differences. Patient-derived fibroblasts remain the most widely used cellular model for biochemical diagnosis and functional characterization of pathogenic variants but cannot recapitulate tissue-specific mechanisms underlying neurological manifestations. Immortalized cell lines facilitate mechanistic investigations, precise genetic manipulation, and high-throughput drug screening, whereas patient-derived iPSCs enable studies of disease-relevant human neural cell types while preserving the complete genetic background of affected individuals.

Recent advances in genome engineering and stem cell biology have further transformed the field. CRISPR/Cas9 technology has enabled the generation of isogenic disease models for establishing causal relationships between pathogenic variants and cellular phenotypes, while directed differentiation of patient-derived iPSCs into neural progenitor cells, neurons, astrocytes, and oligodendrocytes has made it possible to investigate developmental abnormalities directly in human cells. These approaches bridge the gap between reductionist cellular systems and complex in vivo models, providing unprecedented opportunities to dissect cell-type-specific mechanisms of disease. Although three-dimensional brain organoids have revolutionized modeling of numerous neurodevelopmental disorders, analogous models for inherited cholesterol biosynthesis disorders remain largely unavailable, representing an important direction for future research.

In this review, we provide a critical overview of experimental models currently used to investigate inherited disorders of cholesterol biosynthesis, including SLOS, desmosterolosis, lathosterolosis, and CHILD syndrome. We compare genetically engineered animal models, patient-derived fibroblasts, immortalized and genome-edited cell lines, and iPSC-based systems, emphasizing the unique advantages and limitations of each platform for studying sterol metabolism, neurodevelopment, and disease pathogenesis.

## 2. Cholesterol Biosynthesis and Human Disease

Cholesterol is an essential component of cellular membranes and serves as the precursor of numerous biologically active molecules required for normal cellular development and function [7,8]. Its metabolism comprises a complex sequence of biochemical reactions, including the mevalonate pathway followed by post-lanosterol cholesterol biosynthesis [9,10,11]. This process is particularly important during embryogenesis, when cholesterol plays a crucial role in the regulation of morphogenetic signaling pathways, organogenesis, and central nervous system development. Disruption of cholesterol biosynthesis results in an altered sterol profile characterized by reduced cholesterol levels and the accumulation of intermediate sterol metabolites [12,13]. These metabolic abnormalities give rise to a group of congenital disorders characterized by multiple developmental malformations, cognitive impairment, and a broad spectrum of clinical manifestations that vary depending on the affected organs and tissues [6,12,13,14,15,16,17].

### 2.1. Major Steps of Cholesterol Biosynthesis

Cholesterol biosynthesis is a complex multistep process in which each enzymatic reaction contributes to maintaining the balance between the production of the final product and the levels of intermediate sterol metabolites [10,18]. This pathway comprises the mevalonate pathway followed by post-lanosterol transformations, generating numerous intermediate sterols that play important roles in the regulation of cellular processes and are directly implicated in the pathogenesis of inherited disorders of cholesterol metabolism.

The mevalonate pathway constitutes the central metabolic cascade of cholesterol biosynthesis and is localized primarily in the cytosol and endoplasmic reticulum [19]. The pathway begins with acetyl-CoA, which undergoes sequential condensation reactions to form acetoacetyl-CoA and subsequently 3-hydroxy-3-methylglutaryl-coenzyme A (HMG-CoA). The rate-limiting and major regulatory step of the pathway is the reduction of HMG-CoA to mevalonate catalyzed by 3-hydroxy-3-methylglutaryl-coenzyme A reductase (HMGCR), which determines the overall flux through the cholesterol biosynthetic pathway [20].

Mevalonate subsequently undergoes a series of ATP-dependent phosphorylation reactions, yielding mevalonate-5-phosphate and mevalonate-5-diphosphate, followed by decarboxylation to generate isopentenyl pyrophosphate (IPP) [20]. IPP exists in equilibrium with its isomer, dimethylallyl pyrophosphate (DMAPP), thereby providing the activated isoprenoid building blocks required for subsequent biosynthetic reactions [21].

The condensation of IPP and DMAPP gives rise to geranyl pyrophosphate (GPP), which subsequently condenses with an additional isoprenoid unit to form farnesyl pyrophosphate (FPP) [22]. At this stage, the pathway diverges, as FPP serves not only as the precursor for sterol biosynthesis but also as a substrate for the synthesis of numerous non-sterol isoprenoids, including dolichols, ubiquinone (coenzyme Q), and isoprenoid moieties required for protein prenylation.

The condensation of two FPP molecules is catalyzed by squalene synthase, resulting in the formation of squalene [23]. This reaction marks the completion of the pre-squalene stage of cholesterol biosynthesis, which is responsible for the generation of isoprenoid precursors, and the initiation of the post-squalene stage. The latter comprises the conversion of squalene into lanosterol followed by a series of modifications of the sterol nucleus and side chain that ultimately yield cholesterol. Squalene first undergoes epoxidation and subsequent cyclization to produce lanosterol [9]. Lanosterol represents the first cyclic sterol intermediate and serves as the branching point for subsequent enzymatic reactions, including sequential demethylation, isomerization, and reduction steps that ultimately generate cholesterol [10].

Regulation of the mevalonate pathway occurs primarily at the level of HMGCR through sterol regulatory element-binding proteins (SREBPs), as well as via enzyme degradation and feedback inhibition mediated by sterols and non-sterol isoprenoid derivatives [10]. An important feature of this pathway is that its intermediate metabolites, particularly FPP and geranylgeranyl pyrophosphate, serve as substrates for the post-translational prenylation of numerous proteins, including small GTP-binding proteins, thereby linking the mevalonate pathway to the regulation of cell proliferation, intracellular trafficking, and multiple signaling pathways.

The post-lanosterol stage of cholesterol biosynthesis proceeds through two alternatives, partially overlapping pathways: the Kandutsch–Russell pathway and the Bloch pathway [11]. Both pathways originate from lanosterol and comprise a series of sequential reactions involving C14 and C4 demethylation, double-bond reduction, and isomerization of the sterol nucleus and side chain [10].

A characteristic feature of the Kandutsch–Russell pathway is the early reduction of the C24 double bond in the sterol side chain [11]. Following the conversion of lanosterol to zymosterol (ZYM), subsequent reactions generate intermediates including zymostenol (ZYME), lathosterol, and 7-DHC, the immediate precursor of cholesterol [24]. The final reaction is catalyzed by DHCR7, which reduces the C7 double bond to produce cholesterol.

In contrast, the Bloch pathway retains the C24 double bond throughout most of the biosynthetic cascade, with its reduction occurring only during the final step [11,25,26]. Following the formation of ZYM, the pathway proceeds through intermediates including desmosterol, which serves as the immediate precursor of cholesterol in this route. The terminal reaction is catalyzed by DHCR24, which reduces the C24 double bond in the sterol side chain [27].

Although both pathways utilize a common set of enzymatic reactions, including. cytochrome P450 family 51 (CYP51)-dependent demethylation together with multiple reductases and isomerases, they differ in the sequence of sterol side-chain modifications [10,11]. In mammalian cells, the Kandutsch–Russell and Bloch pathways operate in parallel, and their relative contributions depend on tissue type, developmental stage, and metabolic status.

Intermediate sterols, including ZYM, lathosterol, 7-DHC, and desmosterol, possess intrinsic biological activity and modulate multiple cellular processes, including sterol regulatory element-binding proteins (SREBP) signaling and the organization and function of membrane microdomains [28,29]. Accumulation of these sterol intermediates resulting from defects in the corresponding biosynthetic enzymes underlies a number of inherited disorders of cholesterol biosynthesis, highlighting their importance for understanding disease pathogenesis and associated cellular phenotypes [12].

Impaired activity of enzymes involved in either the mevalonate or post-lanosterol stages of cholesterol biosynthesis results in profound alterations of the cellular sterol profile, characterized by cholesterol deficiency accompanied by the accumulation of biologically active sterol intermediates. Given the essential roles of cholesterol and its precursors in embryonic development and cellular homeostasis, inherited defects affecting individual steps of this metabolic pathway are associated with a broad spectrum of congenital developmental disorders [12,13,27]. The following sections summarize the major inherited disorders of cholesterol biosynthesis, including Smith–Lemli–Opitz syndrome, desmosterolosis, lathosterolosis, and CHILD syndrome, with emphasis on their molecular mechanisms, biochemical consequences, and clinical manifestations.

### 2.2. Genetic Disorders of Cholesterol Biosynthesis

**Figure 1:**
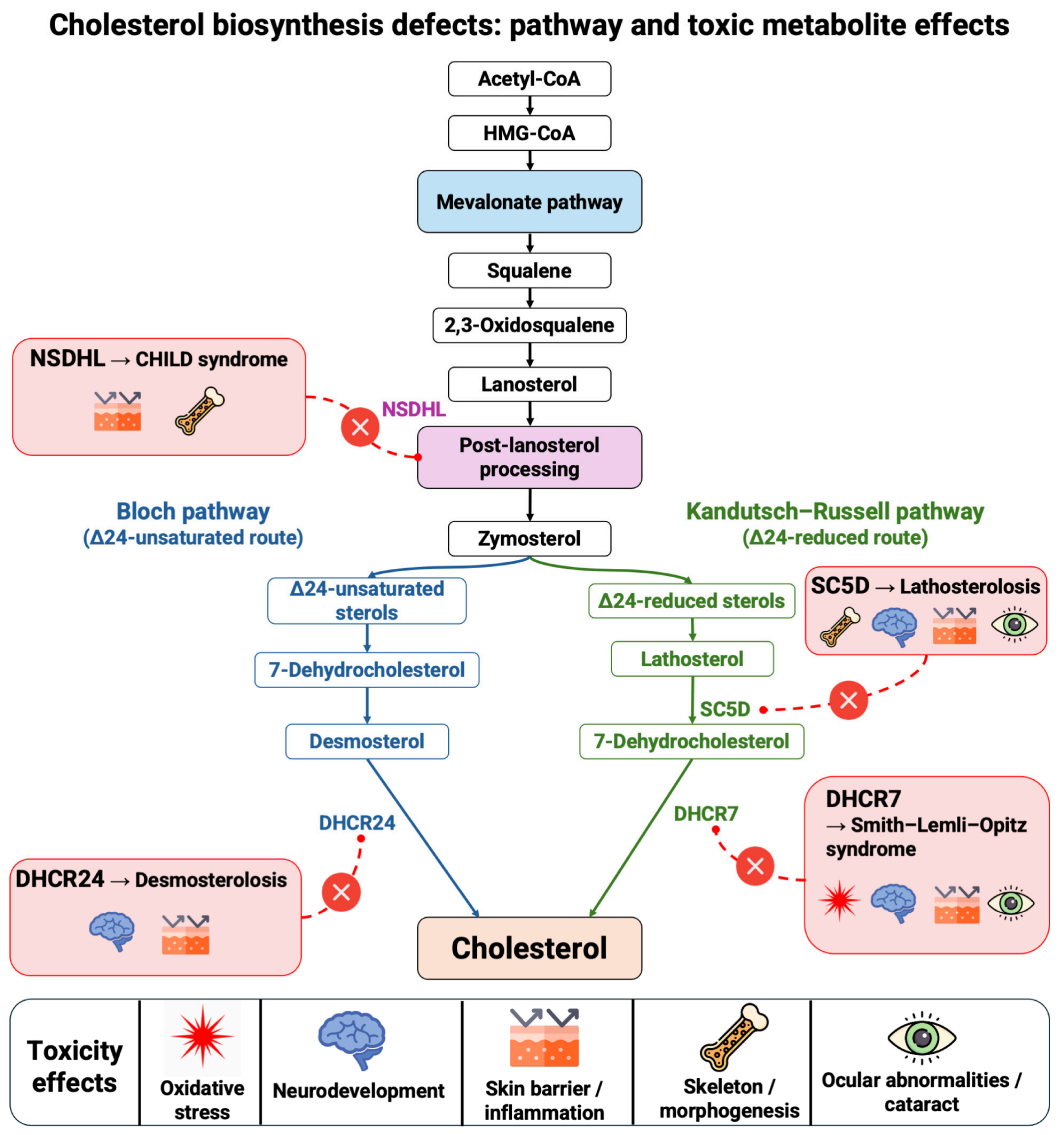
Disorders of cholesterol biosynthesis affecting the post-lanosterol stage. The schematic illustrates the transition from the mevalonate pathway to post-lanosterol cholesterol biosynthesis, including the Bloch and Kandutsch–Russell pathways. Deficiencies of NSDHL, DHCR24, SC5D and DHCR7 disrupt distinct steps of this metabolic cascade, resulting in the accumulation of disease-specific sterol intermediates and the development of inherited disorders of cholesterol biosynthesis. These disorders exhibit varying degrees of involvement of the central nervous system, skin, skeletal and visual system.

Genetic disorders of cholesterol biosynthesis cause a group of congenital diseases in which defects in individual enzymes of the post-squalene stage result in reduced cholesterol synthesis accompanied by the accumulation of disease-specific sterol intermediates. The pathogenesis of these disorders is determined not only by cholesterol deficiency but also by the toxic effects of accumulated sterol metabolites (Figure 1). Smith–Lemli–Opitz syndrome (SLOS) was the first cholesterol biosynthesis disorder to be described and remains the most common disorder within this group. Consequently, other disorders associated with defects in cholesterol metabolism were initially considered either variants of SLOS or part of its diagnostic spectrum [28]. This classification was largely driven by the substantial phenotypic overlap among these conditions. However, as clinical, biochemical, and molecular genetic evidence accumulated, distinct disease-specific features emerged, allowing these disorders to be recognized as separate clinical entities [29].

#### 2.2.1. SLOS

SLOS is a congenital autosomal recessive disorder belonging to the group of inherited defects of cholesterol biosynthesis [28]. The disease is caused by biallelic pathogenic variants in the *DHCR7* gene, located on chromosome 11q13.4 [27]. The gene consists of nine exons and eight introns and encodes the DHCR7 enzyme. Individuals carrying a single pathogenic variant are typically clinically unaffected but are at risk of having a child with SLOS if the other parent also carries a pathogenic *DHCR7* variant.

To date, more than 180 *DHCR7* variants have been reported, of which 65 are classified as pathogenic [27]. These include missense variants, nonsense variants, splice-site variants, and small insertions and deletions that result in reduced enzyme activity or complete loss of function [30]. The most common pathogenic variants include c.964-1G>C, c.452G>A (p.Trp151Ter), c.278C>T (p.Thr93Met), c.976G>T (p.Val326Leu) and c.1054C>T (p.Arg352Trp). Among these, c.964-1G>C, also referred to as IVS8-1G>C, is one of the most prevalent variants in European populations and is associated with the most severe clinical phenotype [31]. The presence of this variant, particularly in the homozygous state, is associated with a more severe clinical course, markedly reduced cholesterol levels, and a high risk of antenatal or early postnatal mortality [27].

The biochemical consequences of *DHCR7* mutations result from disruption of the final step of cholesterol biosynthesis [32]. Excessive accumulation of 7-DHC is particularly detrimental because this sterol is highly susceptible to oxidation, leading to the formation of oxysterols [6]. These oxidation products can damage neuronal and retinal cells, promote oxidative stress, and impair the normal maturation of neuronal populations.

Disruption of the Shh signaling pathway plays a central role in the pathogenesis of SLOS, as this pathway is essential for normal embryonic patterning and the development of midline structures, limbs, the heart, kidneys, lungs, and external genitalia [6]. Cholesterol participates in the regulation of this pathway at multiple levels [33]. First, the Shh protein undergoes post-translational modification, during which cholesterol is covalently attached to the N-terminal signaling fragment. This modification is essential for proper Shh secretion, its incorporation into multimeric complexes, and long-range morphogen signaling. Second, cholesterol is required for the activation of Smoothened (SMO), the key transmembrane component of the Shh pathway in target cells. Under conditions of cholesterol deficiency, both the activation of SMO and its localization to the primary cilium, a microtubule-based organelle on the cell surface that functions as a specialized signaling center, are impaired, resulting in reduced signal transduction to the glioma-associated oncogene homolog family of transcription factors [27].

Reduced Shh signaling accounts for many of the congenital anomalies characteristic of severe forms of SLOS. These include defects of the midline structures, such as holoprosencephaly, craniofacial abnormalities, cleft palate, micrognathia, congenital heart defects, abnormalities of kidney and lung development, and limb malformations, including postaxial polydactyly and syndactyly of the second and third toes [34]. In males, abnormalities of external genital development, including hypospadias and cryptorchidism, are frequently observed. In addition to these somatic manifestations, the disorder is associated with microcephaly, intrauterine and postnatal growth retardation, intellectual disability, delayed speech and motor development, behavioral abnormalities, and features of autism spectrum disorder [6].

Severe SLOS is generally associated with very low cholesterol concentrations and marked elevation of 7-DHC, whereas variants retaining partial DHCR7 activity are more often observed in patients with milder phenotypes [6,27,31,35]. Nevertheless, genotype, residual cholesterol synthesis, biochemical severity, and clinical outcomes do not show a one-to-one relationship, and substantial variability can occur among individuals with similar genotypes. Accordingly, genotype–phenotype associations are informative at the group level but should not be used as deterministic predictors for individual patients.

#### 2.2.2. Desmosterolosis

Desmosterolosis is an extremely rare autosomal recessive disorder of cholesterol biosynthesis, the prevalence of which remains unknown [36]. To date, only a limited number of patients have been reported in the literature, including approximately 14 unrelated cases caused by biallelic pathogenic variants in the *DHCR24* gene [36]. Unlike SLOS, in which the conversion of 7-DHC to cholesterol is impaired due to DHCR7 deficiency, desmosterolosis results from defective reduction of the C24 double bond in sterol intermediates [16]. Consequently, the conversion of desmosterol to cholesterol is impaired, leading to the accumulation of desmosterol in plasma and tissues, particularly in the central nervous system (CNS). Notably, total cholesterol concentrations may remain within or close to the normal range in some patients, complicating the diagnosis in the absence of targeted analysis of intermediate sterols. This feature distinguishes desmosterolosis from several other disorders of cholesterol biosynthesis, in which hypocholesterolemia represents a more pronounced and consistent biochemical hallmark.

The *DHCR24* gene is located on chromosome 1p32.3 and encodes 3β-hydroxysterol Δ24-reductase also referred to as DHCR24. This enzyme belongs to the family of FAD-dependent oxidoreductases and functions during the post-squalene stage of cholesterol biosynthesis [7]. Its primary role is the NADPH-dependent reduction of the C24–C25 double bond of desmosterol to generate cholesterol. In addition, DHCR24 participates in alternative sterol metabolic reactions, accounting for its broader role in sterol homeostasis.

The mutational spectrum of desmosterolosis remains poorly characterized because of the extremely limited number of reported patients [14]. Most pathogenic variants identified to date are missense variants; however, nonsense variants, splice-site variants, and frameshift mutations have also been described. Both homozygous and compound heterozygous genotypes have been reported. One of the most frequently described variants is c.571G>A (p.Glu191Lys), which is located within the highly conserved FAD-binding domain of the protein [16]. This domain is essential for the catalytic activity of the enzyme; therefore, its disruption results in a marked reduction in the ability of DHCR24 to reduce sterol substrates. Other pathogenic variants have also been reported, including c.1412A>G (p.Tyr471Cys) and c.275C>T (p.Thr92Met), identified in the compound heterozygous state [16]. The limited number of documented cases precludes the establishment of robust genotype–phenotype correlations; however, it is generally assumed that patient viability depends on the residual enzymatic activity of DHCR24 and the specific combination of pathogenic alleles [16].

The clinical presentation of desmosterolosis is highly variable and encompasses multiple congenital anomalies, growth retardation, and severe neurodevelopmental impairment [36,37]. The most common manifestations include psychomotor delay, intellectual disability, spasticity, feeding difficulties, failure to thrive, and central nervous system abnormalities, including agenesis or dysgenesis of the corpus callosum, white matter atrophy, ventriculomegaly, seizures, microcephaly, and, less frequently, macrocephaly [14]. These findings underscore the critical role of DHCR24-dependent cholesterol biosynthesis in early brain development. Additional manifestations include craniofacial dysmorphism, such as a high or prominent forehead, low-set and posteriorly rotated ears, micrognathia or retrognathia, and cleft palate, as well as musculoskeletal abnormalities, including distal arthrogryposis, joint contractures, camptodactyly, clinodactyly, syndactyly, limb shortening, and chest wall deformities [37,38,39]. Ophthalmological abnormalities, including nystagmus, strabismus, myopia, and optic nerve atrophy, have also been reported. In some patients, congenital heart defects, cryptorchidism, ambiguous external genitalia, and intrauterine growth restriction have been observed.

In a recent study, Kose et al. reported the first patient with desmosterolosis identified in the Turkish population, thereby expanding the current understanding of the clinical and molecular spectrum of the disorder. The patient carried a previously unreported pathogenic *DHCR24* variant, which was biochemically confirmed by markedly elevated plasma desmosterol concentrations. The clinical phenotype included severe psychomotor developmental delay, profound hypotonia followed by progressive spasticity, feeding difficulties, and multiple dysmorphic features. Neuroimaging revealed central nervous system abnormalities, including agenesis of the corpus callosum. Based on a comparison with previously published cases, the authors identified a recurrent constellation of clinical features characteristic of desmosterolosis. This case highlights the considerable phenotypic variability of the disorder and emphasizes the importance of incorporating sterol profiling together with molecular genetic analysis of *DHCR24* into the diagnostic workup of patients presenting with neurodevelopmental disorders accompanied by congenital anomalies [37].

Desmosterolosis and SLOS are severe congenital disorders of cholesterol biosynthesis associated with high prenatal and neonatal mortality. Although the limited number of reported cases suggests that desmosterolosis may be associated with a particularly severe clinical phenotype, the rarity of the disorder precludes definitive conclusions regarding its relative severity compared with SLOS. It has been proposed that complete loss of DHCR24 activity is frequently incompatible with embryonic development, potentially contributing to the extremely low number of reported living patients [16,36,39].

#### 2.2.3. Lathosterolosis

Lathosterolosis is an ultra-rare autosomal recessive disorder belonging to the group of inherited defects of cholesterol biosynthesis [40]. According to Orphanet, the estimated prevalence is less than 1 case per 1,000,000 individuals. To date, fewer than 20 patients have been reported worldwide, substantially limiting the characterization of the clinical spectrum and the establishment of reliable genotype–phenotype correlations.

In 2002, Brunetti-Pierri et al. demonstrated that the phenotype observed in an affected patient was caused by biallelic pathogenic variants in the *SC5D* gene, resulting in sterol-C5-desaturase deficiency, and established lathosterolosis as a distinct disorder of cholesterol biosynthesis separate from other defects of this metabolic pathway [41]. This enzyme catalyzes the conversion of lathosterol to 7-DHC, representing the penultimate step of cholesterol biosynthesis in the Kandutsch–Russell pathway [7,33]. Consequently, lathosterolosis is pathogenetically closely related to SLOS, as both disorders affect the final stages of cholesterol biosynthesis and are characterized by the accumulation of intermediate sterols.

The genetic basis of lathosterolosis consists of biallelic pathogenic variants in the *SC5D* gene, located on chromosome 11q23.3–q24.1 [29]. Reported pathogenic variants include missense variants, nonsense variants, and splice-site variants that result in loss-of-function of the encoded enzyme. Among the variants described to date are c.212A>T (p.Asn71Ile), c.214C>T (p.Gln72Ter), c.88G>A (p.Arg29Gln), c.137A>C (p.Tyr46Ser) and c.632G>A (p.Gly211Asp) [42]. However, because of the limited number of reported cases, robust genotype–phenotype correlations have not yet been established [29].

The principal biochemical hallmark of lathosterolosis is a marked elevation of lathosterol concentrations in plasma and in tissues, including the cortex, midbrain, liver, kidney, and skeletal muscle [43]. Sterol profiling is of particular diagnostic importance because total cholesterol concentrations may remain within the normal range or be only moderately reduced in some patients [15]. The clinical phenotype of lathosterolosis closely resembles that of SLOS. Common manifestations include psychomotor and intellectual developmental delay, microcephaly, muscular hypotonia, growth retardation, and multiple congenital anomalies [29]. Craniofacial dysmorphism may include sloping forehead, bitemporal narrowing, epicanthal folds, ptosis, a broad nasal bridge, anteverted nares, a long philtrum and micrognathia. Limb abnormalities include postaxial polydactyly, syndactyly of the second and third toes, clinodactyly, and other digital anomalies. This considerable phenotypic overlap with SLOS complicates the clinical diagnosis [15].

One of the most characteristic features of lathosterolosis is ophthalmological involvement, particularly bilateral cataracts, which may progress with age [40]. In contrast to SLOS, lathosterolosis is also more frequently associated with significant liver involvement [29]. Hepatic manifestations range from elevated serum transaminases and cholestasis to fibrosis, cirrhosis, and liver failure. Available clinical observations suggest that the severity of liver disease may be an important determinant of clinical outcome, although the limited number of reported patients precludes definitive conclusions. Severe hepatic involvement may result in an unfavorable prognosis and early mortality, whereas patients with milder liver disease may survive into adolescence and potentially adulthood [42].

Lathosterolosis illustrates why the position of the metabolic block alone is insufficient to predict phenotype. *SC5D* deficiency produces a disease-specific sterol profile and can affect the nervous system, liver, eyes, and skeleton, but the relative contributions of cholesterol deficiency, lathosterol accumulation, and secondary defects in intracellular lipid handling remain unresolved [29,41,42,43,44]. Consequently, experimental models should be evaluated against both biochemical and organ-specific endpoints rather than against a generic cholesterol-deficiency phenotype.

#### 2.2.4. CHILD Syndrome

CHILD syndrome is a rare X-linked dominant disorder belonging to the group of distal cholesterol biosynthesis defects [45]. The classical phenotype is characterized by unilateral involvement of the skin, skeleton, and, in some cases, internal organs, with the lesions typically demonstrating a sharp demarcation at the midline of the body [46].

The disorder is caused by pathogenic variants in the *NSDHL* gene, located on chromosome Xq28 [45]. *NSDHL* encodes decarboxylating sterol-4-alpha-carboxylate 3-dehydrogenase, an enzyme involved in the C4 demethylation of sterol intermediates during the post-squalene stage of cholesterol biosynthesis [14]. The enzyme is localized to the surface of the endoplasmic reticulum and lipid droplets, where it functions as part of a multienzyme complex required for the removal of C4 methyl groups from the sterol backbone.

The mutational spectrum of CHILD syndrome includes missense variants, nonsense variants, splice-site variants, small insertions and deletions, as well as large intragenic deletions involving one or more exons. Molecular genetic analysis of *NSDHL* is currently the primary diagnostic approach, enabling the identification of the majority of pathogenic variants [47]. Robust genotype–phenotype correlations have not been established, most likely because of the unique features of X-linked inheritance and random X-chromosome inactivation in females. Consequently, disease severity is determined not only by the specific *NSDHL* pathogenic variant but also by the proportion and tissue distribution of cells expressing the normal and mutant alleles during embryonic development [14,46]. This concept is supported by recently reported clinical observations. For example, Hettiarachchi et al. (2020) described a female patient carrying a previously unreported heterozygous *NSDHL* variant who presented with the typical unilateral skin lesions and ipsilateral limb abnormalities, including cutaneous syndactyly of the toes [47].

The mode of inheritance is fundamental to understanding the clinical manifestations of CHILD syndrome. The disorder is generally believed to be embryonically lethal in males because individuals with an XY karyotype lack a second X chromosome capable of compensating for *NSDHL* deficiency [48]. As a result, complete loss of enzyme function leads to severe impairment of cholesterol biosynthesis and fetal demise during gestation. The rare reported male patients with a CHILD phenotype are thought to survive primarily because of postzygotic somatic mosaicism [47].

The pathogenesis of the cutaneous manifestations is closely related to the essential role of cholesterol in maintaining the epidermal barrier, cellular membranes, and the intercellular lipid matrix of the stratum corneum [49,50]. Local cholesterol deficiency disrupts keratinocyte differentiation and compromises epidermal barrier function. At the same time, the accumulation of toxic sterol intermediates exerts direct cytotoxic effects on epidermal and dermal cells, promoting inflammation, hyperkeratosis, and the formation of xanthomatous lesions [45].

Accordingly, the hallmark clinical feature of the disorder is the CHILD nevus, a congenital or early postnatal inflammatory ichthyosiform skin lesion [47]. It typically presents as erythematous hyperkeratotic plaques covered with yellowish wax-like scales, showing marked lateralization and a sharp demarcation at the midline. A characteristic feature is ptychotropism, referring to the preferential involvement of major skin folds, including the inguinal and axillary regions [50]. The face is generally spared, although facial involvement has been reported in isolated cases [45]. Cutaneous lesions may be accompanied by pruritus, painful fissures, secondary inflammation, and significant cosmetic disfigurement.

Limb defects are typically observed on the same side of the body as the cutaneous lesions [50]. Their severity ranges from hypoplasia of individual phalanges or digits to severe reduction defects and complete limb aplasia. Additional musculoskeletal manifestations include onychodystrophy, periungual hyperkeratosis, scoliosis, joint contractures, and skeletal malformations. In some patients, the central nervous system, heart, lungs, and kidneys are also affected. This marked phenotypic variability likely reflects differences in the distribution of cell clones carrying the active mutant X chromosome during embryogenesis.

Although the classical presentation of CHILD syndrome is unilateral, bilateral or nearly symmetrical skin involvement does not exclude the diagnosis. In 2026, Zeyrek et al. reported a rare case of bilateral cutaneous involvement in a 7-year-old girl with CHILD syndrome carrying a novel *NSDHL* variant, c.449T>C (p.Phe150Ser) [17]. The authors also demonstrated complete resolution of the skin lesions following topical treatment with a combination of cholesterol and lovastatin [17]. Nevertheless, the true number of bilateral cases remains unknown because of the rarity of the disorder and the absence of a centralized patient registry.

Collectively, inherited disorders of cholesterol biosynthesis constitute a clinically and pathogenetically heterogeneous group of diseases in which defects in individual enzymes disrupt sterol homeostasis [49]. These disturbances are particularly detrimental during embryonic development, when cholesterol is indispensable for membrane biogenesis, developmental signaling, and normal organogenesis [14]. SLOS, desmosterolosis, lathosterolosis, and CHILD syndrome illustrate that defects affecting different steps of post-squalene cholesterol biosynthesis give rise to overlapping but distinct clinical phenotypes. Future research should focus on the development of mechanism-based therapeutic strategies aimed at correcting cholesterol deficiency, reducing the toxicity of accumulated sterol intermediates, and exploring novel pharmacological and gene therapy approaches capable of improving patient outcomes and quality of life.

Among inherited disorders of cholesterol biosynthesis, SLOS has generated the largest body of clinical, biochemical, and experimental evidence and therefore provides the most developed framework for understanding how sterol imbalance affects embryogenesis and neurodevelopment. The combination of 7-DHC accumulation, cholesterol deficiency, impaired Shh signaling, and profound neurodevelopmental abnormalities makes SLOS an informative model for investigating the relationship between sterol metabolism, embryonic morphogenesis, and central nervous system development. However, the marked clinical heterogeneity and complex pathophysiological mechanisms limit the extent to which the disease can be understood solely through clinical observations. Consequently, experimental models capable of reproducing the key biochemical and phenotypic features of SLOS are essential for elucidating its molecular and cellular mechanisms and for the development and evaluation of potential therapeutic strategies. The following chapter reviews the principal experimental models of cholesterol biosynthesis disorders, including animal models, iPSC-based cellular models, and future perspectives for the development of organoid systems.

## 3. Classical and Emerging Models of Cholesterol Biosynthesis Disorders

### 3.1. Animal Models

**Figure 2:**
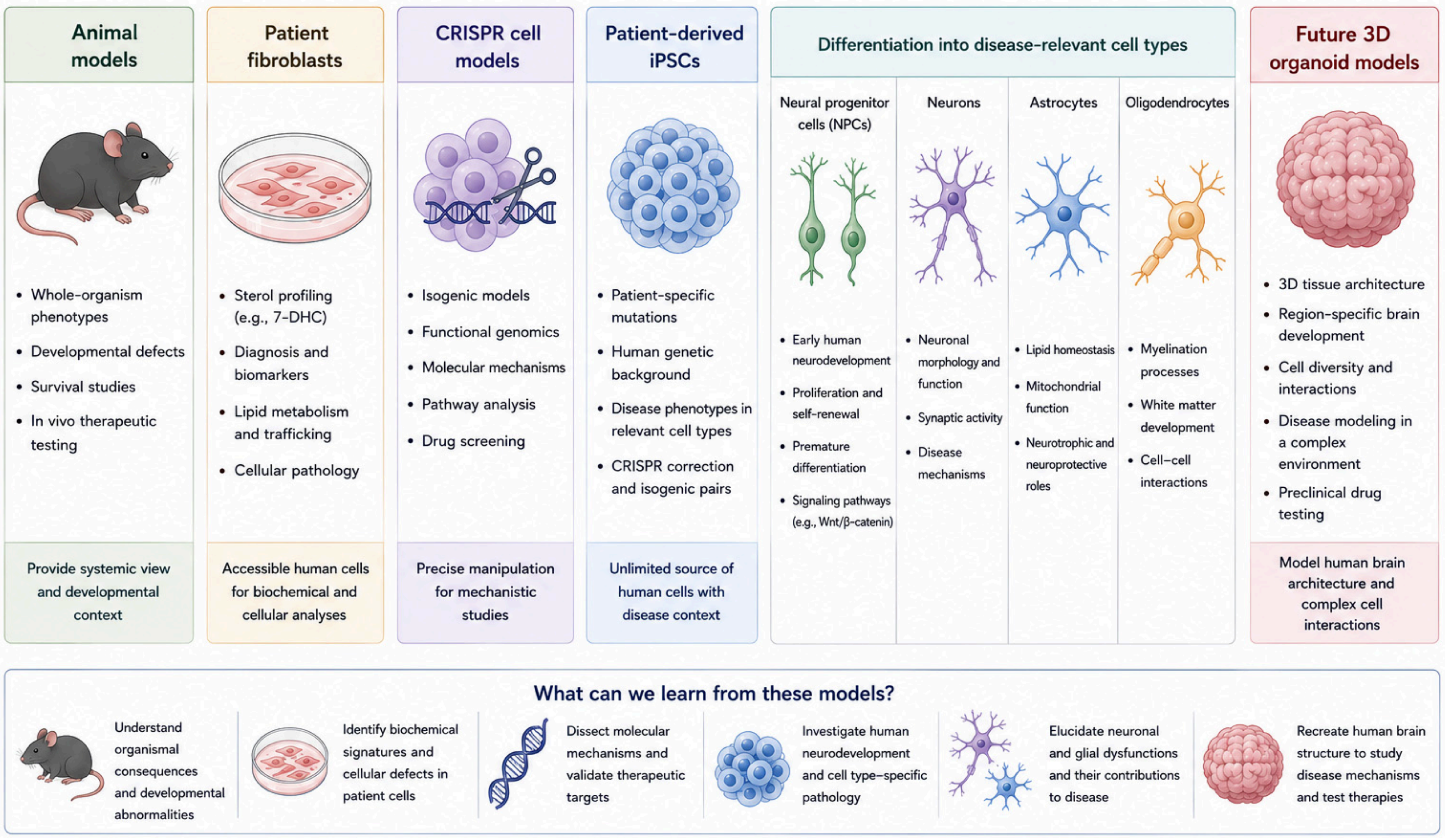
Evolution of experimental models for studying cholesterol biosynthesis disorders. Classical model systems, including animal models, patient fibroblasts, and immortalized cell lines, have provided fundamental insights into sterol metabolism and disease mechanisms. Recent advances in iPSCs technology enable the generation of patient-specific neural progenitor cells, neurons, astrocytes, and oligodendrocytes, allowing investigation of human neurodevelopmental processes directly affected by cholesterol biosynthesis defects. Although organoid models have not yet been established for Smith–Lemli–Opitz syndrome and related disorders, they represent a promising future platform for studying tissue-level pathology and evaluating novel therapeutic approaches.

Classical animal models have played a pivotal role in advancing our understanding of disorders associated with impaired cholesterol biosynthesis (Figure 2). In 2001, Wassif et al. and Fitzky et al. independently generated the first mouse models of SLOS [51,52]. Wassif et al. established a *Dhcr7* knockout mouse by homologous recombination. Postmortem analysis of Dhcr7^−/−^ mice revealed marked accumulation of 7-DHC accompanied by severely reduced cholesterol levels. Perinatal mortality approached 100% within the first 24 h after birth, and the pups exhibited multiple congenital abnormalities, including craniofacial defects, malformations of internal organs, and limb abnormalities closely resembling those observed in infants with SLOS. Neurons isolated from knockout mice retained the ability to generate a single action potential comparable to that of wild-type neurons; however, glutamatergic receptor function was impaired in Dhcr7^−/−^ pups [51].

The model generated by Fitzky et al. was also produced by homologous recombination but differed in the genomic region targeted for disruption. In contrast to the model developed by Wassif and colleagues, homozygous mutant embryos did not exhibit prenatal lethality. Nevertheless, the pups displayed multiple developmental abnormalities, including pulmonary hypoplasia, enlarged urinary bladders, and cleft palate, and all Dhcr7^−/−^ mice died within 18 h after birth because of respiratory failure associated with failure to thrive. Apart from a reduced 8-DHC/7-DHC ratio, the biochemical phenotype closely resembled that observed in patients with SLOS. Notably, Dhcr7^−/−^ pups exhibited *Hmgcr*, *Ldl receptor* and *Srebp-2* mRNA levels comparable to those of heterozygous and wild-type mice; however, total tissue sterol concentrations, HMGCR protein levels and HMGCR enzymatic activity were markedly reduced. Subsequent in vitro studies demonstrated that 7-DHC markedly accelerates HMGCR proteolysis. These findings indicate that, similarly to patients with SLOS, elevated 7-DHC concentrations in *Dhcr7* knockout mice suppress cholesterol biosynthesis, thereby aggravating sterol deficiency and further contributing to abnormal development [52].

A solution to these limitations was proposed by the research group led by F. D. Porter in 2006. The authors generated a mouse model carrying the hypomorphic T93M mutation in the *Dhcr7* gene (commonly referred to as Dhcr7^T93M^) [53]. Unlike the complete knockout model, this mutation preserves partial enzymatic activity, allowing postnatal survival and making the model potentially more relevant for translational studies. Characterization of Dhcr7^T93M/Δ3–5^ mice (harboring one hypomorphic allele and one null allele) revealed syndactyly in 64% of animals and dilation of the second and third cerebral ventricles in 67%. However, biochemical analyses demonstrated that 7-DHC levels in the cerebral cortex, liver, and kidneys declined markedly after 6 weeks of age, while cholesterol concentrations returned to normal. Such biochemical normalization is not observed in patients with SLOS and is likely attributable to species-specific features of murine cholesterol metabolism. Although this model accurately reproduces the hallmark biochemical feature of SLOS, an imbalance between cholesterol and 7-DHC, the absolute sterol concentrations and their ratios differ from those observed in patients. In particular, cholesterol levels generally remain higher, whereas 7-DHC accumulation is less pronounced than in individuals with severe SLOS. These differences most likely reflect interspecies variation in cholesterol metabolism, including a higher rate of cholesterol biosynthesis and the presence of compensatory metabolic mechanisms in rodents. Consequently, this model more closely resembles moderate forms of SLOS than the most severe clinical phenotypes. Thus, the hypomorphic Dhcr7^T93M/Δ3–5^ mouse model has become one of the most widely used experimental systems for SLOS research because it overcomes the perinatal lethality observed in knockout models and enables postnatal studies. Nevertheless, its translational relevance remains limited by species-specific differences in sterol metabolism.

Similar to SLOS, mouse models have also been developed for desmosterolosis through disruption of the *Dhcr24* gene. The Dhcr24^−/−^ mouse model was first generated by Wechsler et al. Newborn Dhcr24^−/−^ mice exhibited body weights approximately 25% lower than those of heterozygous and wild-type littermates. Homozygous mutants accounted for only 10–17% of offspring instead of the expected 25%, indicating partial prenatal lethality. Morphological analysis demonstrated that most organs developed normally, with the exception of the testes, which displayed degenerative changes. In addition, the animals exhibited reduced subcutaneous and visceral fat stores as well as infertility in both sexes [54]. This relatively mild phenotype contrasts with the clinical manifestations reported in human desmosterolosis, although direct comparison is limited by the very small number of described patients. Despite representing a null mutation that completely abolishes cholesterol synthesis, *Dhcr24* deficiency was not lethal because desmosterol is able to partially substitute for cholesterol by incorporating into biological membranes and maintaining their structural integrity, including membrane rigidity [55].

Despite the extreme rarity of lathosterolosis, a mouse model has been developed based on disruption of the *Sc5d* gene. Homozygous Sc5d^−/−^ pups are nonviable and die perinatally, exhibiting intrauterine growth retardation, craniofacial abnormalities (including cleft palate and micrognathia), as well as limb patterning defects [43]. Many of these malformations are consistent with impaired Shh signaling during development, likely resulting from reduced cholesterol levels.

Similar to other inherited disorders of cholesterol biosynthesis, animal models have also been developed for CHILD syndrome. In contrast to SLOS models, the first experimental model of this disorder was generated not by targeted gene disruption but by a spontaneous mutation known as the bare patches (Bpa) mouse, which was subsequently identified as a mutation in the *Nsdhl* gene [56]. A distinctive feature of this model is its X-linked pattern of inheritance: the mutation causes embryonic lethality in hemizygous males, whereas heterozygous females develop pronounced mosaicism as a consequence of random X chromosome inactivation. This results in alternating regions of normal and affected tissue, particularly cutaneous lesions that closely resemble those observed in patients with CHILD syndrome. At the biochemical level, the model is characterized by the accumulation of methylated sterol intermediates, leading to alterations in membrane organization and disruption of signaling pathways involved in morphogenesis.

Cunningham et al. further characterized an *Nsdhl* mutant mouse model of CHILD syndrome, demonstrating tissue mosaicism in heterozygous Bpa1H females that recapitulates the X-linked nature of the disease. In contrast, Bpa1H males exhibited complete perinatal lethality. Owing to random X chromosome inactivation, approximately 50% of tissues were affected in newborn heterozygous females, whereas this proportion decreased to approximately 20% in adulthood. *Nsdhl*-deficient cells were identified in the developing cerebral cortex and retina of female Bpa1H embryos. These findings indicate that although *Nsdhl*-deficient cells in mosaic Bpa1H females are capable of surviving and differentiating during embryonic development, they undergo progressive negative selection throughout postnatal life [57].

Collectively, animal models of inherited disorders of cholesterol biosynthesis have become indispensable tools for investigating the pathogenesis of sterol-dependent diseases; however, their utility depends largely on the nature of the underlying genetic defect and the biological function of the affected enzyme. The most extensively characterized models, particularly those carrying *Dhcr7* mutations, have fundamentally shaped the current understanding of SLOS as a disorder driven not only by cholesterol deficiency but also by the toxic accumulation of intermediate sterols. In contrast, *Dhcr24*-deficient models have revealed a distinct biological phenomenon, namely the ability of desmosterol to partially compensate for cholesterol deficiency, thereby producing a relatively mild phenotype despite complete loss of enzymatic activity. Models carrying *Nsdhl* mutations further illustrate the importance of genetic context, particularly X-linked inheritance, which gives rise to mosaicism and tissue-specific phenotypic variability characteristic of CHILD syndrome.

Despite substantial advances in the development of animal models for monogenic disorders of cholesterol biosynthesis, several fundamental limitations remain. Complete knockout of key biosynthetic enzymes frequently results in embryonic or perinatal lethality, precluding investigation of postnatal disease progression and long-term disease manifestations. Conversely, hypomorphic models permit survival but do not always recapitulate the severe clinical phenotypes observed in patients.

An important limitation common to virtually all animal models of inherited cholesterol biosynthesis disorders is the presence of fundamental interspecies differences in sterol metabolism. Compared with humans, mice exhibit higher rates of endogenous cholesterol synthesis, differences in lipoprotein metabolism, and more efficient compensatory mechanisms that partially preserve sterol homeostasis. In addition, maternal cholesterol transfer during embryogenesis can partially compensate for fetal cholesterol deficiency, thereby attenuating developmental abnormalities and modifying disease severity. Consequently, mouse models often reproduce the biochemical defect but fail to fully recapitulate the clinical spectrum observed in patients, particularly with respect to neurological manifestations and long-term disease progression. These species-specific differences should therefore be carefully considered when interpreting preclinical findings and evaluating the translational potential of candidate therapeutic strategies. Therefore, animal models are most suitable for studying systemic pathophysiology and therapeutic efficacy but should be complemented by human-derived systems when investigating human-specific sterol metabolism and neurodevelopment.

Overall, animal models remain indispensable for investigating systemic pathophysiology and evaluating therapeutic efficacy in vivo. However, species-specific differences in cholesterol metabolism, maternal sterol transfer, and compensatory metabolic pathways limit their ability to faithfully reproduce human disease, particularly neurological manifestations. Consequently, animal models should be regarded primarily as complementary systems rather than definitive predictors of therapeutic responses in patients.

### 3.2. Patient-Derived Cells

Patient-derived cells represent one of the most widely used and reproducible experimental models for investigating inherited disorders of cholesterol biosynthesis. These cells retain the disease-causing genetic defects associated with mutations in *DHCR7*, *DHCR24*, *SC5D* and *NSDHL*, thereby enabling direct analysis of alterations in sterol metabolism, cellular organization, and signaling pathways. Despite the lack of tissue specificity, patient-derived fibroblasts are currently regarded as the reference cellular model for the initial biochemical diagnosis of these disorders and for the functional characterization of pathogenic variants.

Among inherited disorders of cholesterol biosynthesis, patient-derived cell models have been most extensively developed and characterized for SLOS. In the pioneering study by Kelley et al., fibroblasts from patients with SLOS were shown to exhibit a marked accumulation of 7-DHC, reaching approximately 2000-fold above the upper limit of normal, together with a substantial reduction in cholesterol levels. More moderate elevations of 7-DHC were also detected in the plasma and cultured skin fibroblasts of heterozygous carriers, highlighting their potential value as a biochemical diagnostic tool [58]. In subsequent studies, filipin staining, using a fluorescent polyene antibiotic that binds unesterified sterols, demonstrated abnormal sterol accumulation in fibroblasts derived from patients with SLOS. Furthermore, the increased filipin fluorescence and accumulation of lamellar lysosomal inclusions were shown to result from abnormal intracellular accumulation of LDL-derived cholesterol caused by impaired lysosomal degradation rather than from storage of endogenously synthesized 7-DHC. The authors further demonstrated that SLOS fibroblasts were unable to degrade LDL at a normal rate, suggesting that the abnormal LDL-associated cholesterol metabolism represents a toxic consequence of either 7-DHC or one of its metabolites [59].

Subsequent work established patient-derived fibroblasts as a reproducible biochemical model of SLOS. The fraction of newly synthesized sterol converted to cholesterol varied from undetectable to more than 50%, demonstrating allele-dependent residual DHCR7 activity, and lower residual cholesterol synthesis was associated with greater clinical severity at the group level [60]. These data support fibroblasts for functional variant assessment, but they do not imply that fibroblast sterol synthesis alone predicts the neurological phenotype of an individual patient.

Patient-derived fibroblasts have also served as a valuable platform for evaluating therapeutic strategies for SLOS. Treatment of fibroblasts retaining residual DHCR7 activity with simvastatin reduced 7-DHC levels while simultaneously increasing cholesterol synthesis [60]. In an earlier study, Korade et al. demonstrated in SLOS patient fibroblasts that antioxidant therapy consisting of vitamin A, coenzyme Q10, vitamin C, and vitamin E effectively reduced the formation of the major 7-DHC-derived oxysterol, 3β,5α-dihydroxycholest-7-en-6-one. Furthermore, antioxidant treatment normalized multiple disease-associated gene expression changes in SLOS human fibroblasts [61].

More recently, Korade et al. investigated the effects of hydroxyzine (HYZ) as a potential pharmacological approach to reduce excessive 7-DHC accumulation. Human skin fibroblasts derived from patients with SLOS responded to HYZ treatment with a significant reduction in the levels of 7-DHC, 7-dehydrodesmosterol (7-DHD), and dihydrolanosterol (DHL), accompanied by increased levels of zymosterol (ZYM), zymostenol (ZYME), and 8-DHC. Further investigations are needed to establish whether the potentially beneficial effects of lowering 7-DHC, 7-DHD, and DHL levels in SLOS models and patient-derived biomaterials are counterbalanced by increased accumulation of other post-lanosterol sterol intermediates, and whether these changes elicit biological responses comparable to those observed in transgenic mouse models [62]. Collectively, these studies demonstrate that patient-derived fibroblasts provide a robust platform not only for elucidating disease mechanisms but also for evaluating pharmacological strategies aimed at correcting sterol imbalance in SLOS.

In addition to fibroblasts, lymphoblastoid cell lines established from patients’ peripheral blood lymphocytes have also been used to investigate metabolic abnormalities in SLOS. Although this model has been employed far less frequently than fibroblasts, it has confirmed the key features of disturbed sterol metabolism associated with SLOS. Early studies demonstrated that culturing lymphoblastoid cells under conditions of exogenous cholesterol deprivation resulted in a marked increase in the 7-DHC/cholesterol ratio, reflecting reduced DHCR7 activity [63,64]. Moreover, similar, albeit considerably less pronounced, alterations were also observed in heterozygous carriers of *DHCR7* pathogenic variants, with more than 90% of lymphoblastoid cell lines exhibiting an elevated 7-DHC/cholesterol ratio when cultured in sterol-depleted medium. These findings demonstrate the high sensitivity of lymphoblastoid cells to disturbances in cholesterol biosynthesis and confirm the presence of a detectable biochemical phenotype even in the presence of partial DHCR7 activity. Furthermore, studies using lymphoblastoid cells contributed to a better understanding of the mechanisms regulating sterol homeostasis by demonstrating that the extent of 7-DHC accumulation depends on the availability of exogenous cholesterol in the culture medium, indirectly highlighting the importance of compensatory lipoprotein-derived cholesterol uptake. Despite these advantages, lymphoblastoid cell lines have not been adopted as widely as fibroblasts, primarily because they are less suitable for investigating intracellular sterol trafficking, membrane abnormalities, and pharmacological interventions. Consequently, they are currently used mainly for studies of sterol metabolism and functional assessment of DHCR7 activity.

In contrast to SLOS, studies employing patient-derived cells in desmosterolosis remain extremely limited. In the pioneering study by Waterham et al., patient-derived lymphoblastoid cells were used to characterize alterations in sterol metabolism and to identify *DHCR24* as the disease-causing gene. The authors demonstrated marked desmosterol accumulation accompanied by reduced DHCR24 enzymatic activity and substantially decreased cholesterol synthesis. These findings provided evidence that pathogenic variant in *DHCR24*, encoding 24-dehydrocholesterol reductase, underlie the disease. Functional analyses further revealed a profound reduction in DHCR24 activity, confirming the causal relationship between disruption of the final step of cholesterol biosynthesis and the development of the clinical phenotype [65].

A decade later, Schaaf et al. used patient-derived fibroblasts for the molecular genetic and functional characterization of novel DHCR24 variants. These fibroblast cultures enabled transcript analysis and confirmed the pathogenicity of the identified mutations. The study demonstrated that impaired DHCR24 activity was associated with desmosterol accumulation, whereas the cellular phenotype was less pronounced than that observed in SLOS [66]. This difference is likely attributable to the high structural similarity between desmosterol and cholesterol, allowing desmosterol to partially substitute for cholesterol in biological membranes. Consequently, despite the near-complete absence of cholesterol in some cases of desmosterolosis, desmosterol accumulation may partially preserve membrane integrity and function, thereby explaining the relatively mild cellular phenotype compared with disorders characterized by 7-DHC accumulation. Notably, unlike SLOS, no systematic studies investigating intracellular trafficking, oxidative stress, membrane organization, or signaling pathways in patient-derived desmosterolosis cells have been reported to date, highlighting a substantial gap in our understanding of disease pathogenesis.

One of the earliest studies of lathosterolosis was conducted by Brunetti-Pierri et al., who used patient-derived fibroblasts to investigate abnormalities in sterol metabolism. The authors demonstrated that despite the considerable phenotypic overlap between lathosterolosis and SLOS, the biochemical profiles of these disorders differ substantially. Specifically, lathosterolosis is characterized by marked accumulation of lathosterol together with relatively low levels of 7-DHC and higher cholesterol concentrations than those observed in SLOS, reflecting the distinct location of the metabolic block within the cholesterol biosynthetic pathway [41]. These findings demonstrated the important diagnostic value of sterol profiling in patient-derived fibroblasts for the differential diagnosis of inherited disorders of cholesterol biosynthesis.

The diagnostic utility of this approach was further confirmed by Krakowiak et al. [43]. The authors analyzed fibroblasts obtained from a patient who had initially been diagnosed with SLOS; however, sterol profiling revealed marked lathosterol accumulation, accounting for approximately 35% of the total cellular sterol pool compared with 6.9% in healthy controls [43]. These findings led to revision of the original diagnosis and establishment of lathosterolosis, highlighting the importance of patient-derived cellular models for the molecular diagnosis of rare disorders of cholesterol biosynthesis.

In addition to biochemical abnormalities, studies using patient-derived fibroblasts have revealed characteristic ultrastructural alterations. Rossi et al. (2007) demonstrated, by transmission electron microscopy, the presence of lamellar cytoplasmic inclusions in fibroblasts from patients with lathosterolosis, closely resembling those observed in Niemann–Pick disease type C and SLOS [44]. The presence of these structures indicates that lathosterol accumulation is associated with disturbances in intracellular lipid trafficking and lipid homeostasis that extend beyond cholesterol deficiency alone. Thus, despite the limited number of available studies, patient-derived fibroblasts have proven highly informative for both the diagnosis of lathosterolosis and the investigation of its pathogenesis. These findings suggest that lathosterol accumulation is associated not only with characteristic alterations in sterol composition but also with pronounced cellular abnormalities, meaning that fibroblast-based models are widely used for further investigation of this rare disorder.

Studies employing patient-derived cells from individuals with CHILD syndrome are likewise limited. In an early study, Goldyne et al. used fibroblasts isolated from affected skin lesions to investigate disease-associated alterations in cellular metabolism. The authors demonstrated that these cells exhibited reduced proliferative capacity associated with increased production of prostaglandin E2 (PGE2). Furthermore, significant differences were observed between fibroblasts isolated from affected and unaffected skin of the same patient, reflecting the characteristic mosaicism of CHILD syndrome resulting from random X-chromosome inactivation [67].

Subsequently, Emami et al. used patient-derived fibroblasts to investigate ultrastructural abnormalities associated with impaired sterol metabolism. Ultrastructural analysis revealed the accumulation of cytoplasmic lipids, lamellar membrane structures, vacuolated intracellular compartments, and peroxisomal abnormalities, indicating profound disturbances in intracellular lipid homeostasis [68]. These findings provided some of the earliest evidence that *NSDHL* deficiency is associated not only with an altered sterol profile but also with marked morphological abnormalities at the cellular level. Patient-derived fibroblasts were subsequently applied to the molecular diagnosis of the disease. Murata et al. investigated a patient presenting with typical unilateral ichthyosiform skin lesions and hypoplasia of the toes of the left foot. Fibroblasts isolated from the affected skin were used for *NSDHL* sequencing, enabling identification of the pathogenic variant and confirmation of the molecular basis of the disease [69]. These findings demonstrated that fibroblast cultures represent not only a valuable model for investigating disease pathogenesis but also an important tool for the molecular diagnosis of rare disorders of cholesterol biosynthesis.

A significant contribution to the understanding of CHILD syndrome pathogenesis was made by Paller et al., who investigated patient-derived fibroblasts and keratinocytes to examine the distribution of mutant cells within affected tissues and to evaluate the efficacy of topical cholesterol and lovastatin therapy. The characteristic unilateral distribution of lesions in CHILD syndrome was shown to reflect selective elimination of keratinocytes and fibroblasts expressing the mutant allele from the clinically unaffected side of the body. The authors further demonstrated that clinical improvement could be achieved not only by restoring cholesterol levels but also by reducing the accumulation of toxic sterol intermediates through combined treatment with cholesterol and inhibitors of sterol biosynthesis [70]. These findings provided important support for the concept that the pathogenesis of cholesterol biosynthesis disorders is determined not only by deficiency of the end product but also by the toxic effects of accumulated sterol intermediates, consistent with the current understanding of the molecular mechanisms underlying SLOS.

Taken together, studies using patient-derived fibroblasts have provided fundamental insights into the pathogenesis of inherited disorders of cholesterol biosynthesis. Comparative analyses indicate that the cellular phenotype is determined not only by cholesterol deficiency but also by the biological properties of the accumulated sterol intermediates. In SLOS, the toxicity of 7-DHC and its oxidative derivatives appears to play a central role, whereas in desmosterolosis, desmosterol partially compensates for cholesterol deficiency, resulting in a milder cellular phenotype. In contrast, lathosterolosis and CHILD syndrome are characterized by the accumulation of atypical sterols that produce more complex and less well-characterized cellular consequences.

Although patient-derived fibroblasts remain the suitable instrument for biochemical diagnosis and validation of pathogenic variants, their inability to reproduce neural development substantially limits their utility for investigating CNS pathology. Therefore, they are considered biochemical rather than neurodevelopmental models.

### 3.3. Immortalized Cell Lines

Immortalized cell lines and CRISPR/Cas9-based genome editing systems have substantially expanded the experimental toolkit available for investigating disorders of cholesterol biosynthesis, including those associated with mutations in *DHCR7*, *DHCR24*, *SC5D* and *NSDHL*. Unlike primary fibroblasts, these models enable the generation of isogenic knockout and knock-in cell lines, providing a controlled platform for dissecting the contribution of individual enzymes to the regulation of sterol metabolism.

One of the most illustrative examples is provided by CRISPR/Cas9-mediated *DHCR7* knockout in immortalized human cell lines, such as HeLa cells, in which gene inactivation results in the accumulation of 7-DHC. These engineered cell lines provide a standardized platform for investigating the molecular pathogenesis of SLOS and for evaluating potential therapeutic strategies. Similar approaches have also been employed in several recent studies using sgRNA-based CRISPR genetic screens. Genome-wide CRISPR screening in HEK293T cells has demonstrated that enzymes involved in cholesterol biosynthesis, including DHCR7 and SC5D, play opposing roles in the regulation of ferroptosis. Specifically, *DHCR7* deletion results in the accumulation of 7-DHC, which suppresses lipid peroxidation and thereby alters cellular susceptibility to ferroptosis [71,72]. In contrast, *SC5D* deletion notably reversed the suppressive effect of *DHCR7* deficiency on ferroptosis [72]. These findings demonstrate that CRISPR-based models not only recapitulate disease-associated metabolic abnormalities but also enable causal investigation of the biological functions of individual sterol intermediates.

In addition to ferroptosis, immortalized cell lines have been widely used to investigate the toxic effects of 7-DHC accumulation. In a series of studies, Korade et al. demonstrated in *DHCR7*-deficient Neuro2a cells that 7-DHC-derived oxysterols reduce cell viability in both a dose- and time-dependent manner, with some compounds exhibiting biological activity at submicromolar concentrations. The reduction in cell viability resulted from a combination of decreased proliferation and enhanced neuronal differentiation of Neuro2a cells. Furthermore, treatment of control Neuro2a cells with a complex mixture of 7-DHC-derived oxysterols induced gene expression changes similar to those previously observed in *DHCR7*-deficient Neuro2a cells [5]. These findings substantially advanced our understanding of the molecular mechanisms underlying SLOS and highlighted the value of immortalized cell lines for investigating the biological activity of intermediate sterols and their contribution to the pathogenesis of cholesterol biosynthesis disorders.

Skubic et al. investigated *DHCR24*- and *SC5D*-knockout HepG2 cells and demonstrated that disruption of either gene increased the proportion of cells in the G0 phase of the cell cycle and reduced the rate of cell proliferation. Moreover, sterols accumulating in DHCR24- and SC5D-deficient cells, including desmosterol and lathosterol, were shown to exert pro-apoptotic effects and inhibit cellular proliferation [73]. *NSDHL*-knockout HEK293T cells have also been generated; however, they have primarily been used to investigate the role of NSDHL as a regulator of breast tumor migration and invasion rather than as a model of cholesterol biosynthesis disorders. Nevertheless, this cell line may provide a useful platform for future studies aimed at elucidating the biochemical mechanisms underlying CHILD syndrome [74].

Despite their obvious advantages, including high reproducibility and suitability for high-throughput screening, immortalized cell lines lack tissue and cell type specificity, which represents a major limitation for investigating neurodevelopment and the pathogenesis of disorders affecting the central nervous system. Furthermore, the altered metabolism characteristic of transformed cells may influence the physiological effects of sterol intermediates. Consequently, CRISPR-engineered cell lines are best viewed as mechanistic platforms rather than disease models capable of reproducing tissue-specific pathology.

### 3.4. iPSC-Based Models

The development of iPSC technology by Takahashi and Yamanaka (2006) [75] has created new opportunities for modeling inherited human diseases. Unlike fibroblasts, iPSCs enable the recapitulation of early stages of human neurodevelopment, allowing investigation of the consequences of intermediate sterol accumulation in neural cells and the molecular mechanisms underlying the development of neurological abnormalities. Furthermore, advances in genome-editing technologies have made it possible to generate isogenic control lines by either correcting pathogenic variants in patient-derived cells or introducing disease-causing mutations into healthy cells. These approaches substantially improve the reliability of experimental findings by directly linking observed phenotypic alterations to specific genetic defects.

Although the application of iPSC-based models to disorders of cholesterol biosynthesis remains at an early stage compared with other inherited neurological diseases, published studies have already demonstrated their considerable value for investigating neurogenesis, lipid metabolism, and cellular signaling pathways. To date, the majority of studies have focused on SLOS, whereas comparable iPSC-based models for desmosterolosis, lathosterolosis, and CHILD syndrome remain largely unavailable, underscoring the need for further development in this field.

#### Generation and Applications of iPSCs in the Study of Lipid Metabolism Disorders

Since the introduction of iPSC technology, substantial efforts have been directed toward improving the safety, efficiency, and reproducibility of cellular reprogramming. Early protocols relied on integrating retroviral or lentiviral vectors; however, permanent integration of transgenes into the host genome raised concerns regarding insertional mutagenesis and residual transgene expression [75,76]. Consequently, non-integrating approaches have become the preferred strategy for disease modeling and potential clinical applications. Currently, the most widely used methods include Sendai virus vectors, episomal plasmids, and synthetic mRNA-based reprogramming [77,78]. Comparative studies have demonstrated that Sendai virus-mediated reprogramming provides high efficiency while avoiding genomic integration, making it one of the most commonly adopted approaches for generating patient-derived iPSC lines [77].

Compared with fibroblasts and transformed lines, iPSCs permit differentiation into neural progenitors, neurons, astrocytes, oligodendrocytes, hepatocytes, keratinocytes, and other disease-relevant lineages. Their comparative value lies in testing whether a biochemical sterol defect produces a phenotype in the cell type affected clinically. For SLOS, neural progenitors can address proliferation and lineage specification, neurons can address morphology and electrophysiology, and astrocytes can address metabolic support and neuron–glia interactions. The appropriate derivative should therefore be selected from the clinical and mechanistic question rather than from the availability of a generic neural differentiation protocol.

Specifically, reprogramming of patient-derived somatic cells using pluripotency factors enables the generation of iPSCs that retain the underlying genetic defects and can subsequently be differentiated into neurons and glial cells. Francis et al. were among the first to establish iPSC lines from patients with SLOS carrying pathogenic *DHCR7* variants and to differentiate them into NPC. Using this iPSC-based model, the authors demonstrated that the primary driver of SLOS pathogenesis is not cholesterol deficiency per se but the toxic effects of 7-DHC accumulation. Furthermore, they showed that elevated 7-DHC levels impair NPC proliferation and promote precocious differentiation, accompanied by suppression of signaling pathways, including Wnt/β-catenin signaling. Importantly, activation of the canonical Wnt pathway prevented the neural phenotypes observed in SLOS iPSCs. These findings provided evidence supporting a mechanistic link between abnormal sterol metabolism and impaired human neurodevelopment and highlighted the value of iPSC-based models for identifying therapeutic targets that would be difficult to investigate in vivo [79].

More recently, Kawatani et al. expanded the application of iPSC technology beyond neuronal lineages by generating astrocytes from SLOS patient-derived iPSCs. Although astrocytes have historically received less attention than neurons in studies of SLOS, they play essential roles in cholesterol synthesis, metabolic support, synapse formation, and neuronal survival within the central nervous system. The authors demonstrated that SLOS astrocytes exhibit profound disturbances in lipid homeostasis accompanied by mitochondrial dysfunction and impaired oxidative metabolism. Furthermore, these cells displayed reduced neurotrophic and neuroprotective activity, suggesting that glial dysfunction may significantly contribute to neurological manifestations of the disease. These observations support the emerging concept that SLOS should be considered a multicellular disorder involving complex interactions between neurons and glial cells rather than a purely neuronal pathology [80].

Comparable studies remain unavailable for the other disorders of cholesterol biosynthesis; however, the continued development of iPSC technology is highly relevant to all monogenic diseases. Differentiation of iPSCs into neural cell types enables modeling of the early stages of central nervous system development. At the neural progenitor stage, abnormalities in cell proliferation and differentiation can be investigated, whereas differentiated neurons provide an opportunity to examine alterations in neuronal morphology and synaptic function. Furthermore, advances in iPSC technology have paved the way for the generation of three-dimensional organoids, enabling reconstruction of tissue architecture and providing in vitro models that more closely recapitulate the physiological conditions observed in vivo. In contrast to previous model systems, iPSC-derived neural cells combine patient-specific genetics with human neurodevelopment, making them the most physiologically relevant platform currently available for mechanistic studies. Nevertheless, incomplete maturation and limited tissue complexity remain significant challenges.

From a translational perspective, the available experimental systems should be regarded as complementary rather than interchangeable. Animal models remain essential for assessing systemic developmental effects, biodistribution, safety, and in vivo therapeutic efficacy, but species-specific sterol metabolism may limit direct extrapolation to patients. Patient-derived fibroblasts provide a robust platform for confirming biochemical correction and evaluating pharmacological modulation of disease-specific sterol profiles, although they do not reproduce tissue-specific neurological phenotypes. Immortalized and genome-edited cell lines are particularly suitable for mechanistic studies and high-throughput screening but require validation in patient-derived systems. In contrast, iPSC-derived neural and glial cells enable analysis of human cell-type-specific therapeutic responses, although their developmental immaturity and limited representation of systemic physiology remain important constraints. Accordingly, therapeutic development is likely to require a sequential, multimodel strategy in which candidate interventions are first identified in genetically controlled cellular systems, validated in patient-derived cells, and subsequently evaluated in animal models for systemic efficacy and safety.

## 4. Comparative Assessment of Experimental Models

Despite the substantial progress achieved over the past two decades, no single experimental model is capable of reproducing the entire spectrum of pathological processes associated with inherited disorders of post-lanosterol cholesterol biosynthesis. Instead, the currently available experimental platforms should be regarded as complementary systems that address distinct biological questions at different levels of disease complexity. Consequently, the choice of an experimental model should be determined by the specific scientific objective rather than by the assumption that one model is universally superior to another (Table 1).

Animal models remain indispensable for investigating systemic physiology, embryonic development, tissue-specific pathology, and the in vivo efficacy and safety of therapeutic interventions [51,52,53,54,55,56,57]. However, fundamental interspecies differences in cholesterol metabolism, maternal cholesterol transfer during embryogenesis, and compensatory metabolic pathways reduce their ability to fully reproduce the biochemical and neurological manifestations observed in patients [53,81]. Therefore, murine models are particularly valuable for studying organism-level pathophysiology but should ideally be complemented by human-derived experimental systems [53,79,80,81].

Patient-derived fibroblasts continue to represent an established platform for biochemical diagnosis, functional validation of pathogenic variants, and characterization of disease-specific sterol profiles [44,58,59,60,61,62,63,64,65,66,67,68,69,70]. Nevertheless, their inability to model neural differentiation and multicellular interactions substantially limits their application for investigating central nervous system pathology [44,58,59,60,61,62,63,64,65,66,67,68,69,70,79,80]. Similarly, immortalized cell lines and CRISPR/Cas9-engineered systems provide highly reproducible platforms for mechanistic studies, causal investigation of individual genes, and high-throughput drug screening [71,72,73,74]. However, their transformed phenotype and limited cellular complexity reduce their physiological relevance, particularly for disorders affecting neurodevelopment [71,72,73,74].

Among currently available human models, patient-derived iPSCs provide the closest approximation to early human development while preserving the complete genetic background of affected individuals [79,80,82]. Their capacity to generate disease-relevant cell types, including neurons, astrocytes, oligodendrocytes, and other neural lineages, enables investigation of cell type-specific mechanisms that cannot be addressed using conventional cellular models [79,80,85,86]. Furthermore, integration of iPSC technology with genome editing allows the generation of isogenic control lines, thereby substantially reducing genetic background variability and improving mechanistic interpretation [79,82]. Brain organoids further extend these capabilities by partially reproducing the cellular diversity and three-dimensional organization of the developing human brain, although incomplete maturation, cellular stress, and protocol-dependent variability remain important limitations [82,83,84,85,86,87]. To our knowledge, disease-specific organoid models have not yet been reported for SLOS, desmosterolosis, lathosterolosis, or CHILD syndrome.

From a translational perspective, these experimental models should not be viewed as competing alternatives but rather as sequential and complementary components of the therapeutic development pipeline [44,51,52,53,54,55,56,57,58,59,60,61,62,63,64,65,66,67,68,69,70,71,72,73,74,75,76,77,78,79,80]. Modern discoveries obtained in genetically engineered cell lines may be validated in patient-derived fibroblasts and iPSC-based systems, whereas candidate therapeutic strategies, including pharmacological compounds, gene replacement, and genome-editing approaches, require subsequent evaluation in animal models before clinical translation [51,52,53,54,55,56,57,58,59,60,61,62,70,71,72,73,74,75,76,77,78,79,80]. At the same time, disease-specific sterol profiling and emerging lipidomic biomarkers provide a common framework for comparing biochemical phenotypes across experimental platforms and assessing therapeutic efficacy [13,58,59,60,61,62,63,64,65,66,88,89,90,91,92,93].

Taken together, the integration of animal models, patient-derived primary cells, genome-engineered cell lines, iPSC-derived neural systems, and advanced organoid technologies provides the most comprehensive strategy for investigating inherited disorders of post-lanosterol cholesterol biosynthesis. Rather than replacing one another, these experimental platforms address complementary aspects of disease biology and collectively facilitate the translation of mechanistic discoveries into clinically relevant therapeutic strategies [44,51,52,53,54,55,56,57,58,59,60,61,62,63,64,65,66,67,68,69,70,71,72,73,74,75,76,77,78,79,80,82,83,84,85,86,87].

## 5. Current Limitations and Future Perspectives

Despite considerable advances in the study of disorders associated with impaired cholesterol biosynthesis, including SLOS, desmosterolosis, lathosterolosis and CHILD syndrome, currently available experimental models remain insufficiently representative and fail to fully recapitulate disease pathogenesis at the organismal level. These limitations arise from both the biological complexity of these disorders and the technical constraints of existing model systems. Major challenges include the limited physiological relevance of current models, the incomplete understanding of the biological activities and toxic properties of sterol intermediates that accumulate following disruption of cholesterol biosynthesis, and the scarcity of experimental data for ultra-rare disorders such as desmosterolosis and lathosterolosis.

One of the major limitations is the discrepancy between experimental models and the human clinical phenotype. Classical knockout models, particularly those targeting *Dhcr7* and *Sc5d*, frequently result in embryonic or perinatal lethality, precluding investigations of postnatal development and long-term neurological manifestations. Conversely, hypomorphic models permit postnatal survival but do not consistently reproduce the severe forms of these disorders. In the case of NSDHL, model development is further complicated by X-linked inheritance and cellular mosaicism, which complicate data interpretation and hinder the establishment of a universally reproducible experimental model.

Another important limitation is the inability of current in vitro systems to reproduce the complexity of the physiological environment. Although patient-derived fibroblasts remain the gold standard for biochemical diagnosis, they do not capture the unique biological characteristics of central nervous system cell types, which represent the primary targets in many sterol-related disorders. Likewise, immortalized cell lines and CRISPR/Cas9-engineered models, despite their excellent reproducibility and precise genetic manipulation, are limited by the absence of tissue-specific characteristics and their simplified metabolic organization. Consequently, findings obtained using these systems should be interpreted with caution when extrapolating to the organismal level.

The limited understanding of the biological roles of individual sterol intermediates represents another major gap in our knowledge of cholesterol biosynthesis disorders. Although the accumulation of 7-DHC resulting from *DHCR7* deficiency has been extensively characterized, the mechanisms underlying its toxicity, including the contribution of its oxysterol derivatives, remain incompletely understood. Likewise, the physiological and pathological roles of desmosterol in *DHCR24* deficiency and lathosterol in *SC5D* deficiency remain poorly characterized, particularly with respect to their effects on intracellular signaling pathways and cellular differentiation. For *NSDHL* deficiency, the mechanisms by which methylated sterol intermediates disrupt morphogenesis and tissue organization also remain largely unresolved.

An additional challenge is the extremely limited availability of biological material from patients with rare disorders such as desmosterolosis and lathosterolosis. The small number of reported cases and the corresponding scarcity of patient-derived samples substantially restrict opportunities for systematic investigation and validation of experimental models. Consequently, many current conclusions are based on isolated clinical observations or small experimental cohorts, emphasizing the need for additional studies and the development of more representative human disease models.

Sterol profiling represents an important translational link between experimental disease models and clinical practice. Disease-specific sterol abnormalities can be used not only for biochemical diagnosis but also to determine whether an experimental model reproduces the metabolic phenotype observed in patients [13]. In SLOS, increased 7-DHC concentrations and the 7-DHC/cholesterol ratio are established biochemical markers of DHCR7 deficiency and can be quantified in plasma, cultured cells, and prenatal samples [88,89,90]. Analysis of 8-DHC and other non-cholesterol sterols may provide additional information regarding the extent of disruption of the distal cholesterol biosynthetic pathway [13,88]. Moreover, 7-DHC-derived oxysterols may reflect the oxidative consequences of sterol accumulation. In particular, DHCEO has been identified in SLOS fibroblasts and mouse brain and proposed as a marker of 7-DHC oxidation [91]. In desmosterolosis and lathosterolosis, elevated desmosterol and lathosterol, respectively, represent disease-specific biochemical signatures that may remain diagnostically informative even when total cholesterol concentrations are normal or only moderately reduced [13,41,66].

Comparative measurement of these metabolites in patient plasma, primary cells, genetically engineered cell lines, iPSC-derived cells, and animal tissues therefore provides a practical criterion for evaluating the biochemical fidelity of experimental models [83,92]. A model that carries the relevant genetic defect but fails to reproduce the characteristic sterol profile may have limited value for mechanistic or therapeutic studies. Conversely, normalization of a disease-specific sterol ratio or reduction in a toxic oxysterol following treatment may serve as a pharmacodynamic marker, even before changes in complex cellular or clinical phenotypes become apparent [61,62,63,91]. Targeted GC–MS and LC–MS/MS assays enable sensitive quantification of multiple cholesterol precursors and oxysterols [13,92,93], whereas high-resolution and untargeted lipidomic approaches may reveal broader changes in phospholipids, sphingolipids, neutral lipids, and oxidized lipid species. Integration of these measurements with transcriptomic, proteomic, and functional data may facilitate the identification of biomarker panels that connect the molecular response of an experimental model with clinically relevant outcomes. However, broader clinical implementation will require standardized sample preparation, validated reference intervals, harmonization across analytical platforms, and longitudinal studies to determine which individual biomarkers or biomarker combinations reliably reflect disease severity and therapeutic response [13].

At the same time, recent technological advances have created new opportunities to overcome these limitations. One of the most promising approaches is the use of iPSCs. These systems can be differentiated into neurons and glial cells, thereby providing physiologically relevant models for investigating human neurodevelopment and neurodegeneration. In particular, iPSC-derived neurons offer an opportunity to examine the effects of sterol abnormalities on synaptic function, myelination and intercellular communication. Another important direction is the development of three-dimensional organoid models, including cerebral and cortical organoids. These systems recapitulate key aspects of the architecture of the developing human brain and enable investigation of how defects in cholesterol biosynthesis affect neurogenesis, neuronal migration, and cortical layer formation, making them a particularly promising platform for future studies. Such multicellular human models are expected to provide a more physiologically relevant platform for studying disease mechanisms and evaluating novel therapeutic strategies.

Beyond their value for elucidating disease mechanisms, experimental models are becoming indispensable tools for therapeutic development. Patient-derived cells, iPSC-based systems, and animal models provide complementary platforms for evaluating pharmacological compounds, gene correction strategies, and emerging gene therapies, while enabling the identification of pharmacodynamic biomarkers that can facilitate translation from preclinical studies to clinical applications. The integration of these experimental platforms is therefore expected to accelerate the development of targeted therapies for inherited disorders of cholesterol biosynthesis.

Future studies are also expected to benefit from the integration of patient-derived iPSC models with emerging technologies, including single-cell transcriptomics, spatial transcriptomics, high-resolution lipidomics, CRISPR-based genome editing, and artificial intelligence-assisted image analysis. The combination of these approaches will enable comprehensive characterization of cell type-specific sterol metabolism, intercellular interactions, and molecular responses to therapeutic interventions. Such integrated platforms are expected to facilitate biomarker discovery and accelerate the translation of experimental findings into precision therapeutic strategies for inherited disorders of cholesterol biosynthesis.

Combined experimental strategies integrating the strengths of different model systems also hold considerable promise. For example, the application of CRISPR/Cas9 technology to generate isogenic iPSC lines carrying specific pathogenic variants enables direct assessment of allele-specific effects while minimizing the influence of background genetic variation. Likewise, integration of data obtained from animal models and human cellular systems is expected to provide a more comprehensive understanding of the molecular and cellular mechanisms underlying disorders of cholesterol biosynthesis. In parallel, high-resolution metabolomic and lipidomic approaches have emerged as valuable tools for the quantitative characterization of a broad spectrum of sterol intermediates and their downstream metabolites, offering unprecedented opportunities to define disease-specific metabolic signatures and to identify novel therapeutic targets. Single-cell transcriptomics, spatial profiling, and cell-type-resolved lipidomics could determine which cells accumulate particular sterols and how neighboring cells respond, but these technologies should be used to test predefined mechanisms. Organoid studies must additionally address batch variability, stress-associated transcriptional states, diffusion limitations, and incomplete maturation [82,83,84,85,86,87]. Benchmarking against primary fetal references, defined 2D derivatives, and isogenic controls will be essential before subtle disease-associated cell states are interpreted.

## 6. Conclusions

Future progress will require integrated use of genetically engineered animal models, patient-derived fibroblasts, genome-edited cell lines, iPSC-based neural and glial models, organoid technologies, and high-resolution lipidomic and multi-omic analyses. The development of more physiologically relevant experimental models, together with a deeper understanding of the biological functions and pathological effects of individual sterol intermediates, remains one of the major challenges in cholesterol biosynthesis research. Addressing these challenges will be essential not only for elucidating disease mechanisms but also for accelerating the development of targeted therapeutic strategies for inherited disorders of cholesterol biosynthesis.

One of the principal conclusions emerging from this review is that no single experimental model adequately reproduces inherited disorders of cholesterol biosynthesis. Instead, complementary application of animal models, patient-derived cells, genome-edited cell lines, iPSC-based systems, and multicellular organoids is likely to provide the most informative framework for investigating disease mechanisms and developing targeted therapies.

## Figures and Tables

**Table 1:** Experimental models of inherited disorders of post-lanosterol cholesterol biosynthesis: strengths, limitations, and optimal research applications.

Experimental Model	Main Strengths	Major Limitations	Best Application	References
Animal models	Systemic physiology; therapeutic validation	Species-specific differences; early lethality	Pathogenesis, preclinical studies	[43,51,52,53,54,55,56,57,81]
Patient fibroblasts	Sterol profiling; mutation variant validation	Lack of tissue specificity	Biochemical diagnosis, drug testing	[44,58,59,60,61,62,63,64,65,66,67,68,69,70]
Immortalized/CRISPR cell lines	Mechanistic studies (gene function, signaling pathways, sterol metabolism); high-throughput drug and CRISPR screening	Simplified cellular context	Functional genomics	[5,71,72,73,74]
iPSC-derived cells	Human neurodevelopment; patient-specific phenotypes	Incomplete maturation	Disease modeling	[79,80,82]
Brain organoids and multicellular neural cultures	Multicellular CNS architecture	Limited vascularization and maturation. No disease-specific organoid model was identified for these disorders.	Developmental studies; future therapeutic testing	[82,83,84,85,86,87]

## Data Availability

No new data were created or analyzed in this study. Data sharing is not applicable to this article.
